# Patient dumping, outlier payments, and optimal healthcare payment policy under asymmetric information

**DOI:** 10.1186/s13561-016-0135-1

**Published:** 2016-12-20

**Authors:** Tsuyoshi Takahara

**Affiliations:** Graduate School of Economics, Osaka University, 1-7, Machikaneyama, Osaka, 560-0043 Toyonaka Japan

**Keywords:** Patient dumping, Healthcare payment policy, Adverse selection, I13, I18, L51

## Abstract

We analyze a rationale for official authorization of patient dumping in the prospective payment policy framework. We show that when the insurer designs the healthcare payment policy to let hospitals dump high-cost patients, there is a trade-off between the disutility of dumped patients (changes in hospitals’ rent extraction due to low-severity patients) and the shift in the level of cost reduction efforts for high-severity patients. We also clarify the welfare-improving conditions by allowing hospitals to dump high-severity patients. Finally, we show that if the efficiency of the cost reduction efforts varies extensively and the healthcare payment cost is substantial, or if there are many private hospitals, the patient dumping policy can improve social welfare in a wider environment.

## Background

Government agencies in many countries would like to introduce social security systems that decrease healthcare payments while providing high-quality medical services. Two types of healthcare reimbursement policies achieve this end: the retrospective payment system and the prospective payment system.^1^ The retrospective payment system is a cost-based system in which insurers pay the entire treatment cost to hospitals. Under the prospective payment system, insurers pay a fixed amount defined by a government agency for each diagnosis per admission. The latter system incentivizes hospitals to reduce treatment costs and may yield socially optimal cost reductions by hospitals.^2^ Accordingly, some countries have introduced prospective payments to reduce the cost of social security.^3^

Under the prospective payment system, however, hospitals incur risk when treating extraordinarily expensive patients (also called outlier patients). Hospitals that admit outlier patients incur losses even if they make socially optimal efforts to reduce treatment costs. Subsequently, an incentive naturally arises for hospitals to refuse treatment to avoid this financial risk, a phenomenon called the dumping problem in the literature.^4^ The social cost of patient dumping is obvious. For one thing, it triggers potentially fatal treatment delays. Further, as pointed out by Newhouse [25], the dumping problem stimulates patient convergence on particular hospitals, particularly public hospitals, leading to crowding and longer treatment delays.

Given these social costs associated with the possibility of patient dumping, a clear alternative is to insure hospitals for some fraction of the extra costs incurred to treat each outlier patient.^5^ We call this the outlier payment policy for expositional clarity. This policy is precisely what the United States adopted in the 1990s to alleviate the dumping problem. Under this policy, the insurer pays an additional amount equaling some part of the cost exceeding the fixed payment when hospitals admit outlier patients. This additional payment can reduce hospitals’ financial risk, thereby contributing to reduced numbers of dumped patients.

The overall welfare effect of the outlier payment policy is not necessarily clear, however, as gains might arise from allowing hospitals to dump patients at their discretion. We argue that adopting the outlier payment policy is not always justified, even though hospitals are less likely to dump outlier patients when they are insured against such patients. To substantiate this argument, we consider a canonical model of adverse selection in which there are two hospitals, called private and public for expositional clarity. The sole difference between the two is that the insurer may induce the private hospital to dump its patients whereas it cannot allow the public hospital to do so, perhaps because of legal restrictions. We assume that patients randomly visit one of the hospitals that privately observes the treatment cost for each patient.^6^ In this setting, the insurer devises a contract for healthcare payment that is based on the hospital’s level of effort. Given the contract, the hospital decides which patients are to be dumped and decides its level of cost reduction efforts accordingly.

To observe the potential welfare consequences of patient dumping, suppose that the insurer chooses not to adopt the outlier payment policy and instead allows the private hospital to dump high-severity patients selectively. Under this patient dumping policy, it is too expensive for the private hospital to treat high-severity patients; consequently, the bulk of them is eventually transferred to the public hospital. Although patient dumping is in itself welfare-reducing, it also endogenously changes the distribution of patients across hospitals and initiates a sorting effect that substantially alleviates information asymmetry regarding patient types. This sorting effect is potentially welfare-improving because it is instrumental in reducing information rent and consequently in realizing more efficient levels of cost reduction for high-severity patients in equilibrium. We show that gains from the sorting effect can outweigh the social cost of patient dumping under some conditions, suggesting that there are situations in which some degree of patient dumping should be tolerated for the betterment of society.

This study yields several findings. First, if the difference in cost reduction efficiency between high- and low-severity patients is large, the patient dumping policy is optimal in a wider environment, even with a high ratio of high-severity patients. Intuitively, under this circumstance, information rent is large, thus making patient dumping an advantageous payment policy. Second, if healthcare payment cost (administrative cost of a healthcare payment system) is large, the outlier payment policy is preferred over patient dumping, ^7^ because an increase in healthcare payment cost reduces information rent, and therefore, favors outlier payment policy over patient dumping policy. Third, if the number of patients in dumping hospitals (e.g., private hospitals) is large, the patient dumping policy improves welfare to a greater extent. This is because government agencies adopt the patient dumping policy and they need not pay information rent to hospitals that dump high-severity patients, whereas government agencies pay information rent to all hospitals under the outlier payment policy. As such, we insist that insurers consider the circularity of private hospitals when choosing between outlier payment and patient dumping policies.^8^

The main contribution of this study is showing that patient dumping can be an optimal healthcare payment policy. Numerous studies analyze patient dumping (e.g., Newhouse [25]; Dranove [7]; Eze and Wolfe [14]; Newhouse [26]; Meltzer et al. [23]; Canta [4]. Newhouse [25] shows that patient dumping may occur under the prospective payment system and under competition between hospitals. Dranove [7] notes that the patient dumping policy can be efficient due to specialization among hospitals and concentration of patients. Eze and Wolfe [14] also show the optimality of the patient dumping policy using the example of the United States Veterans Affairs hospital inpatient services. Results of these studies parallel ours. However, efficiencies from patient dumping are “gains from specialization”? in both studies, whereas, in the present study, efficiencies from patient dumping are “gains from information acquisition”.

Canta [4] also shows the optimality of the patient dumping policy in a similar environment, considering two types of hospitals. However, our work differs from that of Canta [4] in two points of environment and results. First, Canta [4] does not explicitly analyze the healthcare payment for a public hospital and assumes that a public hospital does not exert a cost reduction effort. In our model, we assume that a public hospital also exerts a cost reduction effort, much like a private hospital, and analyze the effect of the healthcare payment policy on the optimal contract. Through the analysis, we find that the cost reduction effort for high-severity patients and information rent for low-severity patients is greater under the patient dumping policy than under the outlier payment policy. The former effect makes the patient dumping policy advantageous since the distortion is reduced. However, the latter effect makes it disadvantageous since the healthcare payment is increased. Second, Canta [4] assumes that all patients go to the private hospital that can dump high-severity patients and that the public hospital treats only dumped patients. We assume that not all patients go to the private hospital owing to consumer preference or geographical proximity and that a public hospital treats both types of patients. In concrete terms, we assume that a fraction of the patients goes to the private hospital and the remaining go to the public hospital. Although the fraction of patients is given exogenously, in Appendix A. we endogenize the patients’ hospital choice. Our assumption is valid in certain countries where patients can choose hospitals freely. As shown in the analysis, the fraction affects the optimal contract under the patient dumping policy.

We assume that insurers offer severity-dependent contracts to hospitals as a healthcare payment policy, and the severity-dependent contract can be interpreted as outlier payment.^9^ Allen and Gertler [1] discusses the optimality of this selective payment policy.^10^ We assume that hospitals treat patients selectively. This assumption is consistent with the theoretical conclusion of Ellis [10].^11^ Keeler et al. [20] shows that outlier payment acts as insurance for hospitals.^12^ Previous studies also investigate the optimal scheme under outlier payment (Ma [21]; Ellis and McGuire [12]; Jack [18]; Jack [19]; Mougeot and Naegelen [24]. Ellis and McGuire [12] analyzes a consumer-welfare-maximizing outlier payment scheme. Other works study the optimal ratio of outlier payments. Ma [21] investigates optimal outlier payments under the assumption of two-dimensional efforts (cost reduction and treatment quality) by hospitals. He reveals that insurers should reimburse all treatment costs. Moreover, Mougeot and Naegelen [24] studies optimal outlier payment under asymmetric information between insurers and hospitals, and concludes that insurers should reimburse all treatment costs, even under asymmetric information.

## Methods

### Environment

We consider a healthcare payment system in which a public insurer offers contracts to a private and a public hospital for treatment of patients with a specific diagnosis. Throughout the analysis, we denote each hospital by *j*, where *j*=*p* *r* indicates the private hospital and *j*=*p* *u*, the public hospital. As stated, the only difference between them is that the insurer may induce the private hospital to dump patients selectively, whereas it cannot allow the public hospital to do so. The reasoning underlying this assumption is that if a public hospital dumps a specific type of patient, he is unlikely to receive any medical attention; in fact, public hospitals in the United States cannot dump any patient. Apart from this distinction, the two hospitals are assumed to have identical technological acumen.

To simplify, we assume that decision making by patients is given exogenously: *λ*∈(0,1) people select the private hospital, and (1−*λ*) people select the public hospital.^13^ After a patient chooses a hospital, the chosen hospital privately observes patient severity *i*∈{*H*,*L*}, where *i*=*H* denotes a high-severity and *i*=*L*, a low-severity patient. The insurer knows that any given patient is of the high-severity type with probability *ϕ*∈(0,1), which is common knowledge.

### Hospitals

Each hospital can reduce its treatment cost by exerting effort.^14^ The marginal productivity of any cost-reducing effort depends on patient severity, and we assume that cost reduction is higher for low-severity than high-severity patients for the same level of cost reduction efforts. Cost reduction efforts potentially have adverse consequences for hospitals, such as extended duty hours. For a hospital with patient severity *i*, total cost *C*(*i*,*e*) can be written as

1 $$ C(i, e) = c - \theta_{i}e + \frac{1}{2}e^{2}, ~~~~~ i \in \{ L,H\}.  $$

Here, the cost of treatment, *c*, is the same for any patient type and is assumed to be sufficiently large. We also assume *θ*_*L*_>*θ*_*H*_>0, which means that it is easier to reduce the treatment cost for low-severity patients. The last term represents the hospital’s disutility from cost reduction efforts. Letting *w* denote the payment collected from the insurer, the payoff function of a hospital with a type-*i* patient can be written as^15^

2 $$ \tilde\pi (i,w,e) = w - c + \theta_{i}e - \frac{1}{2}e^{2}, ~~~~~ i \in \{ L,H\}.  $$

### Insurer

The insurer offers each hospital a take-it-or-leave-it contract to each hospital that specifies a healthcare payment and a level of cost reduction effort for each patient severity as reported by the hospital. The contract specifies the payment $w^{j}_{\hat i}$ and the level of cost reduction efforts $e_{\hat i}^{j}$ for a given report $\hat i$. Define $\omega ^{j}_{\hat i}\equiv (e^{j}_{\hat i},w^{j}_{\hat i})$ and $\omega ^{j}\equiv ({\omega ^{j}_{H}},{\omega ^{j}_{L}})$.

The insurer seeks to maximize social welfare, assumed to consist of (i) patients’ utility, (ii) the social cost of treatment, and (iii) payment by the insurer. To achieve this, the insurer devises a contract contingent on the hospital’s report $\hat i$ about patient severity. Given the pair of menu contracts (*ω* ^*pr*^,*ω* ^*pu*^), assuming truth-telling, the insurer’s payoff is given by

3 $$ \begin{aligned} W(\omega^{pr},\omega^{pu}) &= \lambda \left\{ \phi \left\{\left[1 - C\left(H,e_{H}^{pr}\right) - \eta w_{H}^{pr}\right](1 - d)\right. \right.\\ &\!\!\!\!\!\!\!\!\!\!\!\!+\left. \left. \left[\gamma \,-\, C\left(H,e_{H}^{pu}\right) - \eta w_{H}^{pu}\right]d\right\} + (1 - \phi)\left[1 - C\left(L,e_{L}^{pr}\right) - \eta w_{L}^{pr}\right]\right\} \\ &\!\!\!\!\!\!\!\!\!\!\!\,+\, (1 \,-\, \lambda)\left\{ \phi \left[1\! - \!C\left(H,e_{H}^{pu}\right) \,-\, \eta w_{H}^{pu}\right] \,+\, (1 - \phi)\left[1 \,-\, C\left(L,e_{L}^{pu}\right) \,-\, \eta w_{L}^{pu}\right] \right\},  \end{aligned}  $$

where *d* is an indicator function that takes *d*=1 when the private hospital dumps high-severity patients and *d*=0 otherwise. We normalize patients’ utility when they receive immediate medical treatment to 1.^16^ In contrast, the utility of dumped patients is given by *γ*∈(−*∞*,1), which captures the ill-effects of patient dumping, such as delayed attention and additional treatment cost.^17^ Finally, *η*∈[1,*∞*) represents a healthcare payment cost.

### Timing

The timing of the game is summarized as follows:

1. the insurer offers contracts to hospitals;
2. a fraction *λ* of patients select the private hospital, and the remaining fraction 1−*λ* of patients select the public hospital, with no patient being aware of his/her severity;
3. the hospitals observe the severity of the patients;
4. they decide whether or not to dump the patients, and if yes, which patients to dump;
5. they set the level of cost reduction efforts;
6. they report patients’ severity to and charge healthcare payments from the insurer, and the contract is implemented.

## Results

### Optimal healthcare payment under symmetric information

This section characterizes the first-best healthcare payment system as a benchmark. With symmetric information, we suppose that the insurer can observe patient severity and thereby can impose its preferred cost reduction efforts on the hospital without information rent. It is easily seen that the insurer prefers no patient dumping, and the first-best contract must satisfy the following participation constraint for each *i*=*L*,*H* and each *j*=*p* *r*,*p* *u*:

$$ {w_{i}^{j}} - c + \theta_{i} {e_{i}^{j}} - \frac{1}{2}{{e_{i}^{j}}}^{2} \ge 0. \qquad\qquad\qquad({P{C^{j}_{i}}}) $$

For each *i*=*L*,*H* and each *j*=*p* *r*,*p* *u*, the insurer’s problem is defined as follows:

4 $$ \begin{aligned} \max_{{\omega_{i}^{j}}} ~ &\lambda\left\{ \phi \left[1 - C\left(H,e_{H}^{pr}\right) - \eta w_{H}^{pr}\right] + (1 - \phi)\left[1 - C\left(L,e_{L}^{pr}\right) - \eta w_{L}^{pr}\right]\right\}\\ &+ (1 - \lambda)\left\{ \phi \left[1 - C\left(H,e_{H}^{pu}\right) - \eta w_{H}^{pu}\right] + (1 - \phi)\left[1 - C(L,e_{L}^{pu}) - \eta w_{L}^{pu}\right] \right\},  \end{aligned}  $$

subject to $\left (PC^{j}_{i}\right)$. All constraints obviously are binding at the optimal solution. Further, there is no reason to treat patients differently as the hospitals are symmetric. This implies that by substituting the participation constraint, the optimization problem for each hospital *j* can be rewritten as

5 $$ \begin{aligned} \max_{{e_{H}^{j}}, {e_{L}^{j}}} ~ & \phi \left[1 - (1 + \eta) \left(c - \theta_{H} {e_{H}^{j}} + \frac{1}{2}{{e_{H}^{j}}}^{2}\right)\right]\\ &\quad+ (1 - \phi)\left[1 - (1 + \eta)\left(c - \theta_{L} {e_{L}^{j}} + \frac{1}{2}{{e_{L}^{j}}}^{2}\right)\right].  \end{aligned}  $$

Solving this optimization problem, we now obtain the first-best allocation. As we assume no disparity in technology, the solution is symmetric between hospitals.

#### **Proposition 1**

In the absence of asymmetric information between the insurer and hospitals, the optimal cost reduction efforts and the optimal cost reduction efforts (the first-best contract) are as follows:

6 $$\begin{array}{*{20}l} &e_{H}^{pr^{FB}} = e_{H}^{pu^{FB}} = \theta_{H}, \end{array} $$

7 $$\begin{array}{*{20}l} &e_{L}^{pr^{FB}} = e_{L}^{pu^{FB}} = \theta_{L}, \end{array} $$

8 $$\begin{array}{*{20}l} &w_{H}^{pr^{FB}} = w_{H}^{pu^{FB}} = c - \frac{1}{2}{\theta_{H}^{2}}, \end{array} $$

9 $$\begin{array}{*{20}l} &w_{L}^{pr^{FB}} = w_{L}^{pu^{FB}} = c - \frac{1}{2}{\theta_{L}^{2}}. \end{array} $$

### Optimal healthcare payment under asymmetric information

#### Optimal outlier payment policy

In this subsection, we obtain the optimal healthcare payment under outlier payments, or simply the optimal outlier payment policy, under asymmetric information. Formally, any outlier payment policy requires the insurer to devise a contract that satisfies all participation constraints. Furthermore, since the insurer cannot observe patient severity, the optimal contract must satisfy the following incentive compatibility constraint for each *i*=*L*,*H* and each *j*=*p* *r*,*p* *u*:

$${w_{i}^{j}} + \theta_{i} {e_{i}^{j}} - \frac{1}{2}{{e_{i}^{j}}}^{2} \ge w_{\tilde{i}}^{j} + \theta_{i} e_{\tilde{i}}^{j} - \frac{1}{2}{e_{\tilde{i}}^{j}}^{2},\ i \neq \tilde{i}.\qquad I{C_{i}^{j}}  $$

Because the insurer designs the payment system to bar patient dumping, *d*=0 in (3), and the insurer’s problem can be written as

10 $$ \begin{aligned} \max_{{\omega_{i}^{j}}} ~ &\lambda \left\{ \phi \left[1 - C\left(H,e_{H}^{pr}\right) - \eta w_{H}^{pr}\right] + (1 - \phi)\left[1 - C\left(L,e_{L}^{pr}\right) - \eta w_{L}^{pr}\right]\right\} \\ + &(1 - \lambda)\left\{ \phi \left[1 - C\left(H,e_{H}^{pu}\right) - \eta w_{H}^{pu}\right] + (1 - \phi)\left[1 - C\left(L,e_{L}^{pu}\right) - \eta w_{L}^{pu}\right] \right\},  \end{aligned}  $$

subject to $\left (PC^{j}_{i}\right)$ and $\left (IC^{j}_{i}\right)$, for each *i*=*L*,*H* and each *j*=*p* *r*,*p* *u*. The following lemma, which is well known in the literature,^18^ is helpful in solving this optimization problem.

##### **Lemma 1**

At the optimal solution, (${PC}_{H}^{pr}$), (${PC}_{H}^{pu}$), (${IC}_{L}^{pr}$), and (${IC}_{L}^{pu}$) are binding.

This lemma implies that the following equations must be satisfied:

11 $$\begin{array}{*{20}l} &{w_{H}^{j}} = c - \theta_{H} {e_{H}^{j}} + \frac{1}{2} e_{H}^{j^{2}},  \end{array} $$

12 $$\begin{array}{*{20}l} &{w_{L}^{j}} = c - \theta_{L} {e_{L}^{j}} + \frac{1}{2} e_{L}^{j^{2}} + {e_{H}^{j}} \Delta \theta,  \end{array} $$

for *j*=*p* *r*,*p* *u*, where *Δ* *θ*≡*θ*_*L*_−*θ*_*H*_>0. Note, also, that the problem faced by one hospital is again independent from and identical to that faced by the other hospital because there is no technology gap in treatment. The optimization problem then can be rewritten as

13 $$ \begin{aligned} \max_{e_{H}^{pr}, e_{L}^{pr}, e_{H}^{pu}, e_{H}^{pu}} ~ &\lambda \left\{ \phi \left[1 - (1 + \eta) \left(c - \theta_{H}e_{H}^{pr} + \frac{1}{2}e_{H}^{pr^{2}}\right)\right.\right. \\ &\left.+ (1 - \phi)\left[1 - (1 + \eta)\left(c - \theta_{L}e_{L}^{pr} + \frac{1}{2}e_{L}^{pr^{2}}\right) - \eta e_{H}^{pr} \Delta \theta \right]\right\} \\ &+(1 - \lambda) \left\{ \phi \left[1 - (1 + \eta) \left(c - \theta_{H}e_{H}^{pu} + \frac{1}{2}e_{H}^{pu^{2}}\right)\right. \right. \\ &\left.+ (1 - \phi)\left[1 - (1 + \eta)\left(c - \theta_{L}e_{L}^{pu} + \frac{1}{2}e_{L}^{pu^{2}}\right) - \eta e_{H}^{pu} \Delta \theta \right]\right\}.  \end{aligned}  $$

Using the above, we obtain the optimal cost reduction effort under the outlier payment policy:

14 $$\begin{array}{*{20}l} &e_{H}^{pr,O*} = e_{H}^{pu,O*} = \theta_{H} - \frac{\eta}{1 + \eta}P\Delta\theta,  \end{array} $$

15 $$\begin{array}{*{20}l} &e_{L}^{pr,O*} = e_{L}^{pu,O*} = \theta_{L},  \end{array} $$

where $P \equiv \frac {1 - \phi }{\phi }$. Here, we assume $\theta _{H} - \frac {\eta }{1 + \eta }P\Delta \theta > 0$ to assure the existence of an interior solution.

Next, we obtain the optimal healthcare payment using (11), (12), (14), and (15). It is straightforward to show that

16 $$\begin{array}{*{20}l} &w_{H}^{pr,O*} = w_{H}^{pu,O*} = c - \frac{1}{2}{\theta_{H}^{2}} +\frac{1}{2} \left(\frac{\eta}{1 + \eta}P\Delta\theta\right)^{2},  \end{array} $$

17 $$\begin{array}{*{20}l} &w_{L}^{pr,O*} = w_{L}^{pu,O*} = c - \frac{1}{2}{\theta_{L}^{2}} + \left(\theta_{H} - \frac{\eta}{1 + \eta}P\Delta\theta\right)\Delta \theta.  \end{array} $$

Comparing (6) and (14), we observe that the level of cost reduction efforts for high-severity patients under the outlier payment policy is distorted downward. Since the level of cost reduction efforts under the outlier payment policy is smaller than the first-best level, the total treatment cost and the optimal healthcare payment for high-severity patients are larger (the third term in (16)). In contrast, the optimal cost reduction efforts for low-severity patients under the outlier payment policy is not distorted, and the insurer needs to set a higher healthcare payment (the third term in (17)). This, too, is an effect of information asymmetry. We summarize the optimal contract under the outlier payment policy as follows.

##### **Proposition 2**

When the insurer constructs the healthcare payment system so as not to dump any patient, the optimal healthcare payment compared to the first-best case is such that

$$\begin{array}{*{20}l} &e_{H}^{pr,O*} = e_{H}^{pu,O*} < e_{H}^{pr^{FB}} = e_{H}^{pu^{FB}},\\ &e_{L}^{pr,O*} = e_{L}^{pu,O*} = e_{L}^{pr^{FB}} = e_{L}^{pu^{FB}},\\ &w_{H}^{pr,O*} = w_{H}^{pu,O*} > w_{H}^{pr^{FB}} = w_{H}^{pu^{FB}},\\ &w_{L}^{pr,O*} = w_{L}^{pu,O*} > w_{L}^{pr^{FB}} = w_{L}^{pu^{FB}}. \end{array} $$

We denote the optimized social welfare in the case of the outlier payment policy as $W^{O^{*}}$.

#### Optimal patient dumping policy

We now examine the optimal payment policy when the private hospital is induced to dump high-severity patients. In this case, the insurer sets the healthcare payment for the private hospital with a participation constraint with respect to low-severity patients only. The insurer then obviously offers the first-best contract for low-severity patients. The profit of the private hospital when it admits high-severity patients is given by

18 $$ \pi (H, e_{L}^{FB}) =w_{L}^{FB} - c + \theta_{H}e_{L}^{FB} - \frac{1}{2}e_{L}^{FB} = \theta_{L}(\theta_{H} - \theta_{L}) < 0.  $$

Hence, the private hospital would refuse to treat high-severity patients, which can be observed by the insurer. The insurer’s objective function then can be written as

19 $$ \begin{aligned} \max_{{\omega_{i}^{j}}} ~ &\lambda \left\{ \phi \left[\gamma \,-\, C\left(H,e_{H}^{pu}\right) - \eta w_{H}^{pu}\right] + (1 - \phi)\left[1 - C\left(L,e_{L}^{pr}\right) - \eta w_{L}^{pr}\right]\right\} \\ \!+ &(1\!\! -\! \lambda)\left\{ \phi \left[1\!\! - \!C\left(H,e_{H}^{pu}\right) \!- \eta w_{H}^{pu}\right] \,+\, (1 \!- \phi)\left[1\! - C\left(L,e_{L}^{pu}\right) \!- \!\eta w_{L}^{pu}\right] \right\},  \end{aligned}  $$

subject to (${PC}_{L}^{pr}$), (${IC}_{i}^{pu}$), and (${PC}_{i}^{pu}$), for each *i*=*L*,*H* and each *j*=*p* *r*,*p* *u*. Note that the public hospital still is barred from patient dumping, and thus, the participation constraint for high-severity patients must be satisfied for the public hospital.

To solve this problem, we reapply Lemma 1 and obtain

20 $$\begin{array}{*{20}l} &w_{L}^{pr} = c - \theta_{L} e_{H}^{pr} + \frac{1}{2} e_{L}^{pr^{2}},  \end{array} $$

21 $$\begin{array}{*{20}l} &w_{H}^{pu} = c - \theta_{H} e_{H}^{pu} + \frac{1}{2} e_{H}^{pu^{2}},  \end{array} $$

22 $$\begin{array}{*{20}l} &w_{L}^{pu} = c - \theta_{L} e_{L}^{pu} + \frac{1}{2} e_{L}^{pu^{2}} + e_{H}^{pu} \Delta \theta.  \end{array} $$

Given these, the problem can be rewritten as

23 $$ \begin{aligned} \max_{e_{L}^{pr}, e_{H}^{pu}, e_{L}^{pu}} ~ &\lambda (1 - \phi)\left[1 - (1 + \eta)\left(c - \theta_{L}e_{L}^{pr} + \frac{1}{2}e_{L}^{pr^{2}}\right)\right] \\ &\!+ \!(1\! - \!\lambda)(1 \,-\, \phi)\left[1 \!- \!(1 + \eta)\left(c \!- \theta_{L} e_{L}^{pu} \,+\, \frac{1}{2}e_{L}^{pu^{2}}\right) - \frac{1}{2} \eta e_{H}^{pu} \Delta \theta \right]\\ &+ \phi \left[\lambda\gamma + (1-\lambda) - (1 + \eta)\left(c - \theta_{H}e_{H}^{pr} + \frac{1}{2}e_{H}^{pr^{2}}\right)\right].  \end{aligned}  $$

This problem yields the optimal cost reduction efforts under a patient dumping policy:

24 $$\begin{array}{*{20}l} &e_{L}^{pr,D*} = \theta_{L},  \end{array} $$

25 $$\begin{array}{*{20}l} &e_{H}^{pu,D*} = \theta_{H} - (1 - \lambda)\frac{\eta}{1 + \eta}P\Delta\theta,  \end{array} $$

26 $$\begin{array}{*{20}l} &e_{L}^{pu,D*} = \theta_{L}.  \end{array} $$

Further, the optimal healthcare payment can be obtained by substituting (24), (25), and (26) into (20), (21), and (22):

27 $$ w_{L}^{pr,D*} = c - \frac{1}{2} {\theta_{L}^{2}},   $$

28 $$ \begin{aligned} w_{H}^{pu,D*} &= c - \frac{1}{2}\left[\theta_{H} - (1 - \lambda)\frac{\eta}{1 + \eta}P\Delta\theta\right]\\ &\qquad\left[\theta_{H} + (1 - \lambda)\frac{\eta}{1 + \eta}P\Delta\theta\right],  \end{aligned}  $$

29 $$ w_{L}^{pu,D*} \,=\, c \,-\, \frac{1}{2}{\theta_{H}^{2}} \,+\, \left[\theta_{H} \,-\, (1 \,-\, \lambda)\frac{\eta}{1 + \eta}P\Delta\theta\right]\Delta \theta.   $$

Unlike previous cases, the optimal contract in this case is asymmetric between hospitals even though we assume no asymmetry in technology. The key is that the insurer need not provide information rent to the private hospital but still pays it to the public hospital. Comparing (17) and (29), we observe that information rent for the public hospital is higher under the patient dumping policy than under the outlier payment policy. In contrast, comparing (14) and (25), we also show that distortion in the level of cost reduction efforts for high-severity patients is smaller under the patient dumping policy than under the outlier payment policy. The optimal healthcare payment system in the case of the patient dumping policy is summarized by the following proposition.

##### **Proposition 3**

The optimal healthcare payment policy under the patient dumping policy is such that

$$\begin{array}{*{20}l} e_{L}^{pr,D*} = e_{L}^{pu, D*} = &e_{L}^{pr, O*} = e_{L}^{pu, O*} = e_{L}^{pr^{FB}} = e_{L}^{pu^{FB}}, \\ e_{H}^{pu,D*} > &e_{H}^{pu,O*} > e_{H}^{pu^{FB}},\\ w_{L}^{pr, O*} > &w_{L}^{pr, D*} = w_{L}^{pr^{FB}},\\ w_{H}^{pu, O*} > &w_{H}^{pu, D*} > w_{H}^{pu^{FB}}, \notag\\ w_{L}^{pu, D*} > &w_{L}^{pu, O*} > w_{L}^{pu^{FB}}. \end{array} $$

We denote the optimized social welfare in the case of the patient dumping policy as $W^{D^{*}}$.

### Welfare analysis

#### Welfare comparison

We thus far have characterized optimal contracts under two distinct regimes: outlier payment and patient dumping. Given the indicated results, we now are ready to compare social welfare between the two policies to illustrate whether a degree of patient dumping should be tolerated and, if so, under what conditions. To this end, we first compute the welfare difference between the two policies (hereafter, welfare difference) as follows:

30 $$\begin{array}{*{20}l} W^{D*} - W^{O*} &= \underbrace{\frac{1}{2}\phi\lambda(1 + \eta)\left(\frac{\eta}{1 + \eta}P\Delta\theta\right)^{2}(2 - \lambda)}_{\text{Heavy-severity patients}}  \\ &\underbrace{- \phi\lambda (1 - \gamma)}_{\text{Patient dumping cost}}  \\ &\underbrace{+ \lambda(1 - \phi)\eta(\theta_{H} - \frac{\eta}{1 + \eta}P\Delta\theta)\Delta\theta}_{\text{Low-severity patients in the private hospital}}  \\ &\underbrace{- (1 - \lambda)(1 - \phi)\eta\lambda\frac{\eta}{1 + \eta}P\Delta\theta^{2}}_{\text{Low-severity patients in the public hospital}}.  \end{array} $$

Obviously, the patient dumping policy is preferred over the outlier payment policy when this difference is strictly positive. The first term gives the welfare difference associated with the treatment cost and the payment cost when *i*=*H* (which for expositional simplicity we call the welfare difference for high-severity patients). As mentioned, the optimal level of cost reduction efforts for high-severity patients is higher under the patient dumping policy, which always contributes to welfare improvement. The second term gives the welfare difference associated with patients’ utility when *i*=*H* and *j*=*p* *r* (the welfare difference for high-severity patients in the private hospital). It is always negative because the utility of dumped patients is discounted to *γ*. The third term gives the welfare difference when *i*=*L* and *j*=*p* *r* (the welfare difference for low-severity patients in the private hospital). It is always positive because the insurer need not pay information rent to the private hospital under the patient dumping policy. Finally, the last term reflects the welfare difference when *i*=*L* and *j*=*p* *u* (the welfare difference for low-severity patients in the public hospital). It is negative because the insurer must provide a larger information rent in this contingency under the patient dumping policy.

The patient dumping policy clearly is less likely to be optimal when its cost is relatively large (*γ* is small). We can subsequently conjecture that there is a threshold level $\bar \gamma $ such that the outlier payment policy is optimal if and only if $\bar {\gamma } > \gamma $. By rearranging (30), the threshold is computed as

31 $$ \begin{aligned} \bar{\gamma} &= 1 - \underbrace{\frac{1}{2}\frac{\eta^{2}}{1 + \eta}P^{2}\Delta\theta^{2}(2 - \lambda)}_{\text{High-severity patients}} - \underbrace{\eta P(\theta_{H} - \frac{\eta}{(1 + \eta)}P\Delta\theta)\Delta\theta}_{\text{Low-severity patients in the private hospital}} \\&\qquad+ \underbrace{(1 - \lambda)P^{2}\frac{\eta^{2}}{1 + \eta}\Delta \theta^{2}}_{\text{Low-severity patients in the public hospital}}\\ &= 1 - \eta P\Delta\theta\left(\theta_{H} - \frac{\eta}{1 + \eta}P\Delta\theta\left(1 - \frac{1}{2}\lambda\right)\right).  \end{aligned}  $$

Since $\theta _{H} - \frac {\eta }{1 + \eta }P\Delta \theta > 0$ and *λ*∈(0,1) by assumption, $\theta _{H} - \frac {\eta }{1 + \eta }P\Delta \theta (1 - \frac {1}{2}\lambda) > 0$. We also assume *η* and *Δ* *θ* are positive, and by definition *P* is positive. This implies that $\bar {\gamma } < 1$. We then obtain the following result, which is not surprising by itself but still clarifies that the patient dumping policy can be optimal under some conditions.^19^

##### **Proposition 4**

There is a threshold patient dumping cost $\bar {\gamma }$ that satisfies $\bar {\gamma } \in (-\infty, 1)$.

### The optimal cost reduction efforts and the information rent analysis

We now assess the impact of changes in external conditions on the optimal healthcare payment system via the optimal level of cost reduction efforts and the change in information rent. We particularly focus on changes in the ratios of low-/high-severity patients and public/private hospital patients.

#### Higher ratio of high-severity patients

We begin with the effect of the ratio of low-/high-severity patients, as captured by *P*, and examine how a change in *P* affects the threshold $\bar {\gamma }$. A change in *P* generally has three effects on differences in social welfare. The first effect is the number effect, that is the effect on the number of patients for which the insurer pays information rent to the hospital. If the number of high-severity patients rises, the number of patients for whom the insurer pays information rent to the hospital declines. The second effect is the distortion effect, which is shown in (14) and (25). It can be seen that the extent of distortion in the level of cost reduction efforts diminishes as the number of high-severity patients rises. The third effect is the information rent effect, which is shown in (17) and (29). The magnitude of the information rent shrinks with an increase (decrease) in the number of high-severity (low-severity) patients.

To evaluate the impact on welfare of a change in *P* more precisely, it is instructive to decompose welfare differences into three elements as above: the welfare difference for (i) high-severity patients, (ii) low-severity patients in the private hospital, and (iii) low-severity patients in the public hospital. Using algebraic techniques, we obtain

32 $$ \begin{aligned} -\frac{\partial \bar{\gamma}}{\partial P} = \underbrace{\frac{\eta^{2}}{1 +\eta}P\Delta\theta^{2}(2 - \lambda)}_{\text{High-severity patients~~}} &\underbrace{+\eta[\theta_{H} - 2\frac{\eta}{1 + \eta}\Delta\theta P]\Delta\theta}_{\text{~~Low-severity patients in the private hospital}} \\ & \underbrace{-2(1 - \lambda) P\frac{\eta^{2}}{1 + \eta}\Delta\theta^{2}}_{\text{Low-severity patients in the public hospital}}.  \end{aligned}  $$

1. For high-severity patients, only the distortion effect influences welfare. As seen in (14) and (25), the distortion effect is larger under the outlier payment policy. As such, if *P* decreases (i.e., there are many high-severity and few low-severity patients), the gap in welfare under the two cases shrinks, and $\bar {\gamma }$ increases (shown by the first term in (32)).
2. For low-severity patients in the private hospital, the number effect and information rent effect are influential. If there are many high-severity patients, the number of patients for whom the insurer pays information rent to the private hospital is small. This effect moves $\bar {\gamma }$ upward, since it increases social welfare under the outlier payment policy; however, this effect does not affect social welfare under the patient dumping policy. In contrast, the information rent effect moves $\bar {\gamma }$ downward since it is milder under the outlier payment policy. Hence, if the number effect is weaker than the information rent effect, a decrease in *P* moves $\bar {\gamma }$ downward. When the information rent per patient is smaller (i.e., $\theta _{H} - \frac {\eta }{1 + \eta }P\Delta \theta $ is small), the number effect is weaker (shown by the second term in (32)).
3. For low-severity patients in the public hospital, only the information rent effect is applicable. As seen in (17) and (29), this effect is weaker under the outlier payment policy, which moves $\bar {\gamma }$ downward.

The overall welfare impact of the patient dumping policy is determined by these tradeoffs. In particular, one crucial factor yields the difference in efficiency of cost reduction efforts between the two patient types. We summarize this observation as follows.

##### **Proposition 5**

There exists $\bar {\theta }_{H}$ such that if $\theta _{H} > \bar {\theta }_{H}$, $\frac {\partial \bar {\gamma }}{\partial P} \ge 0$, and if $\theta _{H} < \bar {\theta }_{H}$, $\frac {\partial \bar {\gamma }}{\partial P} \le 0$.

This result asserts an important policy implication. From Proposition 5, there exists a possibility of welfare improvement by abolishing the outlier payment policy even when the number of high-severity patients is large. If the variance in efficiency of cost reduction efforts is large (i.e., *θ*_*H*_ is small against *Δ* *θ*), the patient dumping policy is preferred over the outlier payment policy for more diseases (i.e., $\bar {\gamma }$ moves downward).

**Fig. 1:**
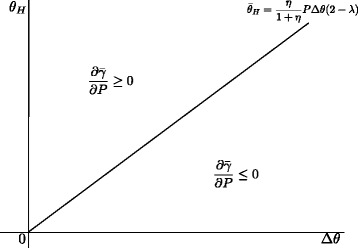
Effect of *P* on $\bar {\gamma }$

Figure 1 depicts the region in which $\frac {\partial \bar {\gamma }}{\partial P} < 0$ holds. The region tends to shrink as healthcare payment cost *η* increases. Intuitively, if the healthcare payment cost is large, the optimal information rent is smaller, and the number effect weakens.

##### **Proposition 6**

$\bar {\gamma }$ rises as the healthcare payment cost *η* increases when *P* is high.

Woolhandler and Himmelstein [33] and Woolhandler and Himmelstein [34] empirically investigated per capita administrative cost for US and Canadian healthcare payment programs and found that it is higher in the United States than in Canada. As such, our model implies that the patient dumping policy is more advantageous in Canada.

#### Higher number of patients in the private hospital

We now examine how a change in the proportion of patients who visit the private hospital, as captured by *λ*, affects the threshold. To this end, it is straightforward to obtain

33 $$ \frac{\partial\bar{\gamma}}{\partial \lambda} = -\frac{1}{2}\frac{\eta^{2}}{1 + \eta}P^{2}\Delta\theta^{2} \le 0.   $$

Note that a change in *λ* does not affect social welfare under the outlier payment policy since the optimal contract is symmetric between the two hospitals and thus independent of *λ*. This is not the case under the patient dumping policy, however, as the optimal contract is asymmetric. If the number of patients who select the private hospital increases (i.e., *λ* increases), the distortion in the cost reduction efforts decreases under the patient dumping policy (25). This moves $\bar \gamma $ upward, thereby favoring the patient dumping policy over the outlier payment policy. However, as shown in (28), the optimal information rent increases as *λ* increases, which yields a countervailing effect and moves *λ* downward. We can show that this latter effect is generally stronger than the former; hence, an increase in *λ* always reduces $\bar \gamma $ as shown in (33).

##### **Proposition 7**

$\bar {\gamma }$ decreases as *λ* increases.

It is intuitively clear that patients’ initial choice of hospitals, given exogenously in our model, could influence the optimal healthcare payment scheme significantly. According to the proposition, under the assumption that patient dumping is allowed only for private hospitals, social welfare can be improved by abolishing the outlier payment policy against many diseases in areas where more private hospitals operate. In practice, this implies that a regulatory agency should admit selected regional variations in the healthcare payment scheme as the number of private institutions is expected to be high in urban areas and low in rural areas.

## Discussion and conclusions

The main result of this paper is that there are cases in which insurers should not reimburse additional payments to hospitals that admit expensive patients even though doing so may trigger socially expensive patient dumping. A payment scheme that insures against outlier patients exacerbates the extent of information asymmetry between insurers and hospitals and consequently results in less-efficient effort for cost reduction. When this cost is sufficiently significant, insurers should instead allow hospitals to dump expensive patients to specific hospitals as a second-best alternative to the outlier payment policy. We show that such a payment scheme, which tolerates a degree of patient dumping, can ease information asymmetry and improve efficiency under some conditions.

One important limitation of our model is the assumption that patients are allocated randomly to hospitals. However, as we have shown in Appendix A, relaxing this assumption does not alter our main contention in any qualitative way. The analysis would be more complete, though certainly more complicated, if we more explicitly modeled patients’ choice of hospitals taking technology difference between hospitals or reputation into consideration. In the future, it might be of interest to explore three-way interactions among insurers, hospitals, and patients.

## Endnotes

^1^For instance, see Newhouse [26] for this classification.

^2^Stephan and Berger [32] noted that a patient’s pathway (preclinical medical plan) shortens her hospital stay and reduces total treatment cost.

^3^For instance, the United States adopted the prospective payment system in 1983. Some countries, notably Japan, still adopt the retrospective payment system.

^4^This problem was discussed in Ma [21].

^5^If insurers pay all of the treatment cost, hospitals take no risk; such a healthcare payment system is equivalent to the retrospective payment system.

^6^This assumption of asymmetric information is common in the literature. See Chalkley and Malcomson (1998), Sappington and Lewis (1999), Glazer and McGuire [16], Beitia [3], Marchand et al. [22], Chalkley and Khalil [5], Siciliani [30], and, Mougeot and Naegelen [24].

^7^Woolhandler and Himmelstein [33] and Woolhandler and Himmelstein [34] compare the administrative cost of healthcare programs per capita between the United States and Canada. Skinner et al. [31] investigates the determinants of inefficiency in the Medicare program in the United States.

^8^The Emergency Medical Treatment and Active Labor Act prohibits patient dumping in any region of the United States, and the Centers for Medicare & Medicaid Services, a division of the United States’ Department of Health and Human Services, reimburse treatment cost under the Medicare program (this can be considered an outlier payment).

^9^For instance, when insurers define the payment for average-severity patients as the fixed payment under the prospective payment system, the difference of payment for high- and average-severity patients can be interpreted as outlier payment.

^10^Numerous studies examine the optimality of outlier payments: Ellis and McGuire [11], Selden [29], Newhouse [26], Chalkley and Malcomson (1998), Ellis [10], Keeler et al. [20], Glazer and McGuire [16], Glazer and McGuire [17], Meltzer et al. [23], Barros [2], Eggleston [9], Jack [19].

^11^See also Ellis and McGuire [13], Eggleston (2000), Frank et al. [15], and Siciliani [30].

^12^See also Marchand et al. [22].

^13^We assume that each patient does not know his or her severity and chooses a hospital based solely on exogenous factors such as proximity. Of course, we can obtain qualitatively similar results as long as exogenous factors have some effect.

^14^For example, the degree of preventive care by doctors can be interpreted as such a variable.

^15^All propositions hold so long as the hospital’s payoff function has the Spence–Mirrlees single crossing property.

^16^In Appendix B. we investigate that the patient dumping policy can be optimal even it has the effect on the treatment quality in the public hospital.

^17^Additional treatment cost includes the social cost indicated by Newhouse [25].

^18^For example, see Salanie [27].

^19^We so far assume that the private/public patient ratio *λ* is unaffected by the insurer’s policy choice. In the Appendix, we show that the following proposition is satisfied even when we patients choice hospital endogenously.

## Appendix A: Endogenous hospital choice

In Section 5, we assume that the private/public patient ratio *λ* is exogenously given and not affected by policy change since the patients cannot recognize the severity of their condition. However, if the insurer introduces the patient dumping policy and the patients are aware of it, a fraction of patients who select the private hospital under the outlier payment policy may select the public hospital *ab initio* to ensure that they are not dumped and do not to bear the dumping cost even when they do not know the severity of their condition.

Further, in the appendix, we build a Hotelling model that describes the patients’ hospital choice. In this model, patients choose a hospital while taking into account the risk of dumping. Lastly, we show that Proposition 4 is satisfied even in this case.

We consider a market where the patients are distributed horizontally and uniformly on the line with length 1. Their location is denoted by *x* where *x*∈[0,1]. The private hospital is located on the left end of the line and the public hospital on the right. The utility of a patient located at *x* and treated at hospital *i* is given by

$$U(x) =\left\{ \begin{array}{ll} v - tx & (i = pr) \\ v - t(1 - x) & (i = pu) \end{array} \right. $$

where *v* is the benefit of treatment provided by the hospitals under the outlier payment policy and *t*(>0) is the marginal transportation cost. Assume that *v* is large enough and all patients on the line are treated at the hospital. There is no technology gap between the two hospitals, and under the outlier payment policy the value of treatment is the same between the hospitals. The location of the patient who is indifferent between the two hospitals under the outlier payment policy is

$$\bar{x} = \frac{1}{2}. $$

Then, we obtain that the fraction of patients who go to the private hospital is $\bar {\lambda } = \frac {1}{2}$ under the outlier payment policy when we endogenize the patients’ hospital choice. Next, we derive the fraction of patients under the patient dumping policy. Under this policy, high-severity patients who go to the private hospital are dumped to the public hospital. Then, the expected utility of patients who go to the private hospital is reduced to *v* ^′^ where 0<*v* ^′^<*v*. Under the patient dumping policy, the location of the patient who is indifferent between the two hospitals is

$$\tilde{x} = \frac{1}{2} - \frac{v - v'}{2t}. $$

Then, we obtain the fraction of patients who go to the private hospital under the patient dumping policy as $\tilde {\lambda } = \frac {1}{2} - \frac {v -v'}{2t}$. Obviously, $\tilde {\lambda } < \bar {\lambda }$, and we find that the patient dumping policy reduces the number of patients who go to the private hospital.

Increasing the proportion of patients who select the public hospital under the patient dumping policy has three effects.^20^ The first is the self-selection effect. Under the outlier payment policy, the expected patient dumping cost is *ϕ* *λ* *γ*. However, if the fraction of patients who select the private hospital under the outlier payment policy select the public hospital first, the expected patient dumping policy is reduced to $\phi \tilde {\lambda }\gamma $. Then, the relative superiority of the patient dumping policy becomes substantial. The second is the optimal contract effect. As seen in (25), (26), (28) and (29), the optimal contract for the public hospital depends on *λ*. As previously demonstrated, in the optimal contract, the cost reduction efforts for high-severity patients and the information rent for low-severity patients are higher under the patient dumping policy than under the outlier payment policy. If *λ* changes to $\tilde {\lambda }$ under the patient dumping policy, these effects weaken. The third is the number effect. Under the patient dumping policy, the optimal information rent for low-severity patients in the private hospital is reduced to zero. Then, if *λ* changes to $\tilde {\lambda }$, this information rent-saving effect weakens due to the change in the number of patients, and the superiority of the patient dumping policy diminishes.

Next, we aggregate these effects. We term the optimized social welfare under the patient dumping policy when a fraction of $\tilde {\lambda }$ select the private hospital as $\tilde {W}^{D^{*}}$. We compute the welfare difference between the two policies as follows:

34 $$ \begin{aligned} \tilde{W}^{D^{*}} - W^{O^{*}} &= \underbrace{\frac{1}{2}\tilde{\lambda}\frac{\eta^{2}}{1 + \eta}\frac{(1 - \phi)^{2}}{\phi}\Delta\theta^{2}}_{\text{High-severity patients}}\\ &\underbrace{- \phi\tilde{\lambda}(1 - \gamma)}_{\text{Patient dumping cost}} \\ &\underbrace{+ (1 - \phi)\bar{\lambda}\eta\left(\theta_{H} - \frac{\eta}{1+\eta}P\Delta\theta\right)\Delta\theta}_{\text{Low-severity patients in the private hospital}} \\ &\underbrace{\,-\, (1 \!- \!\phi)\eta\Delta\theta\left[(\tilde{\lambda} \,-\, \bar{\lambda})\left(\theta_{H} \!- \!\frac{\eta}{1\! + \!\eta}P\Delta\theta\right) \,+\, \tilde{\lambda}(1 \,-\, \tilde{\lambda})\frac{\eta}{1 \,+\, \eta}P\Delta\theta\right]}_{\text{Low-severity patients in the public hospital}}.  \end{aligned}  $$

We denote the threshold *γ* as $\tilde {\gamma }$ between the two policies when patient movement occurs. By rearranging (34), we obtain

35 $$ \begin{aligned} \tilde{\gamma} &= 1 - \left(\frac{1}{2} + \tilde{\lambda}\right)\frac{\eta^{2}}{1 + \eta}P^{2}\Delta\theta^{2} \\&\quad- \left(\theta_{H} - \frac{\eta}{1 + \eta}P\Delta\theta\right)P\eta\Delta\theta\left(2\frac{\bar{\lambda}}{\tilde{\lambda}} - 1\right) \end{aligned}  $$

The second and third terms are negative by assumption. And we obtain that there exists a threshold patient dumping cost $\tilde {\gamma }$ that satisfies $\tilde {\gamma } \in (-\infty, 1)$ even when patient movement occurs.

## Appendix B: Congestion effect

In the analysis, we assume that the patients who are treated immediately can obtain constant utility, which is normalized to 1. However, it is conceivable that under the patient dumping policy, a concentration of patients to the public hospital occurs, and the treatment quality provided by the public hospital changes. It will be worse off if the patient concentration imposes a burden on the physicians in the public hospital, where they cannot refuse the treatment for the high-severity patients, considering skilled physicians often shift to private hospitals. However, it will be better if the increase in the number of patients under the patient dumping policy trains the physicians’ skill in the public hospital.

In this appendix, we investigate this congestion effect on the welfare and optimal healthcare payment policy under the patient dumping policy. Assume that the utility of a patient treated in the public hospital under the patient dumping policy is reduced by *α*. That is, the utility of patients who are treated immediately is 1−*α*, and the utility of patients who are dumped by the private hospital is *γ*−*α*. If *α*>0, the congestion effect improves the treatment quality in the public hospital under the policy, otherwise, *α*<0. Obviously, when *α*>0, the patient dumping policy becomes advantageous.

Next, we show the condition under which the patient dumping policy is preferred to the outlier payment policy. We denote the optimized social welfare under the patient dumping policy with the congestion effect as $\hat {W}^{D*}$. The welfare difference between the two policies is as follows.

36 $$ \begin{aligned} \hat{W}^{D*} - W^{O*} &= \frac{1}{2}\phi\lambda(1 + \eta)\left(\frac{\eta}{1 + \eta}P\Delta\theta\right)^{2}(2 - \lambda) - \phi\lambda (1 - \gamma)\\ &\quad+ \lambda(1 - \phi)\eta\left(\theta_{H} - \frac{\eta}{1 + \eta}P\Delta\theta\right)\Delta\theta\\&\quad- (1 - \lambda)(1 - \phi)\eta\lambda\frac{\eta}{1 + \eta}P\Delta\theta^{2} \\ & \quad - (1 - \lambda + \lambda\phi)\alpha \end{aligned}  $$

The last term indicates the welfare loss due to the congestion effect under the patient dumping policy. We term the threshold *γ* as $\hat {\gamma }$ between the two policies, considering the congestion effect and we obtain

$$\begin{aligned} \hat{\gamma} &= 1 - \eta P\Delta\theta\left(\theta_{H} - \frac{\eta}{1 + \eta}P\Delta\theta\left(1 - \frac{1}{2}\lambda\right)\right) \\&\quad+ \frac{(1 - \lambda + \lambda\phi)\alpha}{\phi\lambda}. \end{aligned} $$

If $\hat {\gamma } < 1$, the following inequality is satisfied.

$$\frac{\eta P\Delta\theta\left(\theta_{H} - \frac{\eta}{1 + \eta}P\Delta\theta\left(1 - \frac{1}{2}\lambda\right)\right)\phi\lambda}{(1 - \lambda + \lambda\phi)} > \alpha $$

If the inequality is satisfied, the patient dumping policy is preferred even when there is the congestion effect under the patient dumping policy. Considering the parameter constraints, the left hand side is negative. Therefore, if the congestion has negative effect on the treatment quality in the public hospital under the patient dumping policy, then the patient dumping policy can be optimal.
